# Novel antifungal agents in clinical trials

**DOI:** 10.12688/f1000research.28327.2

**Published:** 2022-01-12

**Authors:** Samantha E. Jacobs, Panagiotis Zagaliotis, Thomas J. Walsh

**Affiliations:** 1 Division of Infectious Diseases, Icahn School of Medicine, New York, NY, 10029-5674, USA; 2 Transplantation-Oncology Infectious Diseases Program, Division of Infectious Diseases, Department of Medicine, Weill Cornell Medicine, New York, NY, 10065, USA; 3 Departments Pediatrics and Microbiology & Immunology, Weill Cornell Medicine, New York, NY, 10065, USA

**Keywords:** Antifungal Agents, novel treatments, pharmacokinetic and pharmacodynamic, clinical trials

## Abstract

Invasive fungal diseases due to resistant yeasts and molds are an important and increasing public health threat, likely due to a growing population of immunosuppressed hosts, increases in antifungal resistance, and improvements in laboratory diagnostics.  The significant morbidity and mortality associated with these pathogens bespeaks the urgent need for novel safe and effective therapeutics.  This review highlights promising investigational antifungal agents in clinical phases of development: fosmanogepix, ibrexafungerp, rezafungin, encochleated amphotericin B, oteseconazole (VT-1161), VT-1598, PC945, and olorofim.  We discuss three first-in-class members of three novel antifungal classes, as well as new agents within existing antifungal classes with improved safety and tolerability profiles due to enhanced pharmacokinetic and pharmacodynamic properties.

## Introduction

Invasive fungal diseases (IFDs) are a growing public health concern in an expanding population of immunocompromised hosts ^1^. Indeed, attributable mortality may still approach 90% in the most vulnerable patients infected with highly-resistant pathogens ^1^. Three classes of antifungal drugs are currently available for prevention and treatment of IFDs: triazoles, polyenes, and echinocandins. However, use of these agents is often hampered by drug toxicity, drug-drug interactions, and lack of oral formulation. Furthermore, novel therapeutic options are needed due to increasing rates of antifungal resistance and increasing IFDs due to emerging pathogens, many of which are resistant to approved antifungal agents. Herein, we review the antifungal pipeline for agents in clinical phases of development. We give particular attention to investigational drugs with novel mechanisms targeting cellular and biochemical pathways. 

## Agents targeting the cell wall

### Fosmanogepix

***Mechanism of action*.** Glycosylphosphatidylinositol (GPI)-anchored mannoproteins are one of the major cell wall components of fungi. Inhibition of GPI-anchored protein biosynthesis therefore has the potential to compromise cell wall integrity and restrict fungal growth. Fosmanogepix (previously APX001 and E1210; Eisai Company, Japan) is a first-in-class antifungal prodrug that inhibits the fungal Gwt1 (GPI-anchored wall protein transfer 1) gene that encodes a new acyltransferase involved in an early step of the GPI post-translational biosynthetic pathway ^2^. Fosmanogepix undergoes rapid and complete metabolism by systemic phosphatases to its active moiety, manogepix. The chemical structure, mechanism of action, spectrum of activity, clinical trials status, and potential advantages of fosmanogepix and other investigational antifungal agents included in this review are provided in Table 1.

**Table 1.** Novel investigational antifungal agents in clinical trials.

Class	Novel agent	Mechanism of action	Spectrum of activity	Completed/ongoing phase 2 and 3 clinical trials	Potential advantages
Glycosylphos- phatidylinositol (GPI) inhibitors	Fosmanogepix (APX001) 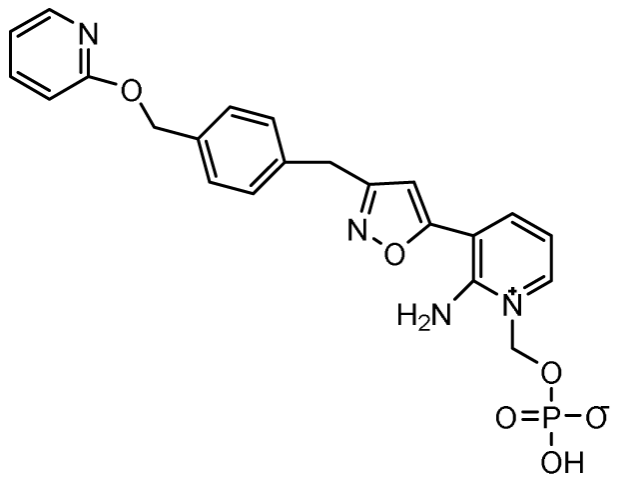	Inhibits the fungal enzyme Gwt1 to disrupt GPI-anchor post-translational protein modification	*Candida* spp. except *C. krusei* *Cryptococcus* spp. *Aspergillus* spp. *Fusarium* spp. *Scedosporium* spp. *Lomentospora prolificans* *Purpureocillium lilacinum* *Rhizopus arrhizus* *Coccidioides* spp.	Ongoing • Treatment of IFIs due to *Aspergillus* spp or rare moulds (NCT04240886) • Treatment of candidemia or invasive candidiasis due to *C. auris* (NCT04148287) • Treatment of candidemia in non-neutropenic patients (NCT03604705)	Broad spectrum and active against highly resistant fungi
Triterpenoids	Ibrexafungerp (SCY-078) 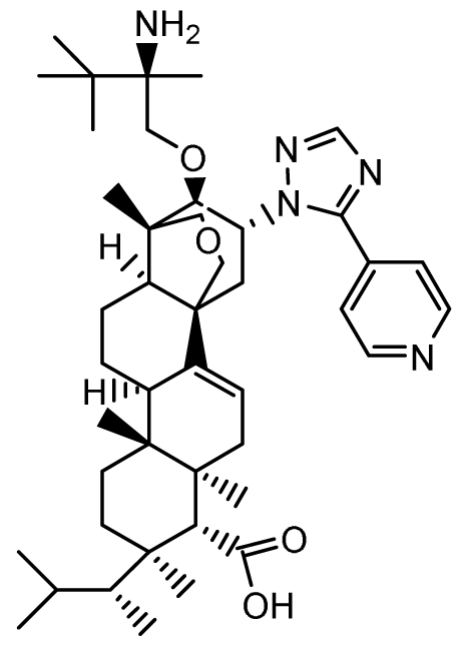	Inhibits (1(3)- β-D- glucan synthase	*Candida* spp. including echinocandin- resistant *C. glabrata* and *C. auris* *Aspergillus* spp. *Paecilomyces variotii* *Pneumocystis jirovecii*	Completed • Step-down therapy for candidemia and/or invasive candidiasis (NCT02244606) • Treatment of acute VVC (DOVE, NCT03253094; VANISH-303, NCT03734991; NCT02679456) Ongoing • Treatment in patients with refractory or intolerant fungal diseases (FURI, NCT03059992) • Ibrexafungerp and voriconazole combination for treatment of invasive pulmonary aspergillosis (NCT03672292) • Treatment of *Candida auris* infection (CARES, NCT03363841) • Prevention of recurrent VVC (CANDLE, NCT04029116) • Treatment of acute VVC (Vanish 306, NCT03987620)	• Active against resistant *Candida* species • First orally bioavailable inhibitor of (1(3)- β-D-glucan synthase • Combination therapy against invasive aspergillosis • Oral fungicidal therapy against *Candida* spp., including step-down for candidemia
Echinocandins	Rezafungin (CD101) 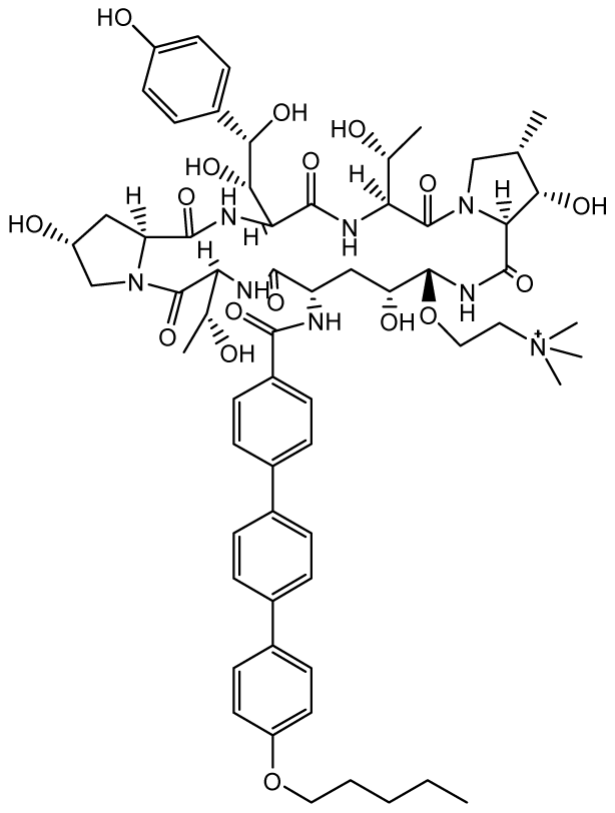	Inhibits (1(3)- β-D- glucan synthase	*Candida* spp. *Aspergillus* spp. *Pneumocystis jirovecii*	Completed • Treatment of candidemia and/or invasive candidiasis with fluconazole stepdown (STRIVE, NCT02734862) • Treatment of acute moderate to severe VVC (RADIANT, NCT02733432) Ongoing • Treatment of candidemia and/or invasive candidiasis (ReSTORE, NCT03667690) • Prevention of invasive fungal disease in patients undergoing allogeneic HCT (ReSPECT, NCT04368559)	• Long half-life allows once weekly dosing; • Not hepatotoxic • Activity may prevent *Pneumocystis* pneumonia
Polyenes	Encochleated amphotericin B (MAT2203) 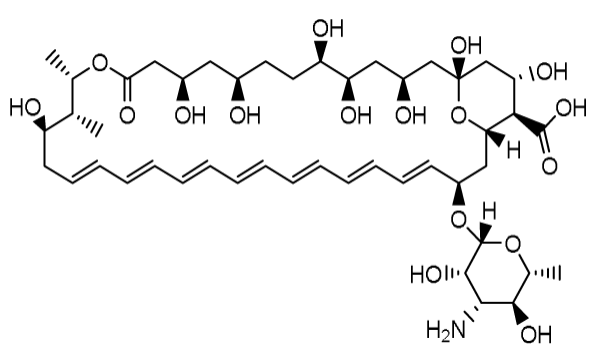	Binds to ergosterol to form pores in fungal cell membrane	*Candida* spp. *Aspergillus* spp. *Cryptococcus* spp.	Completed • Treatment of VVC (NCT02971007) Ongoing • Treatment of refractory mucocutaneous candidiasis (NCT02629419) • Treatment of cryptococcal meningitis in HIV- infected patients (EnACT, NCT04031833)	• Oral formulation • Less toxicity than deoxycholate and lipid formulations of amphotericin B
Tetrazoles	Oteseconazole (VT-1161), VT-1598 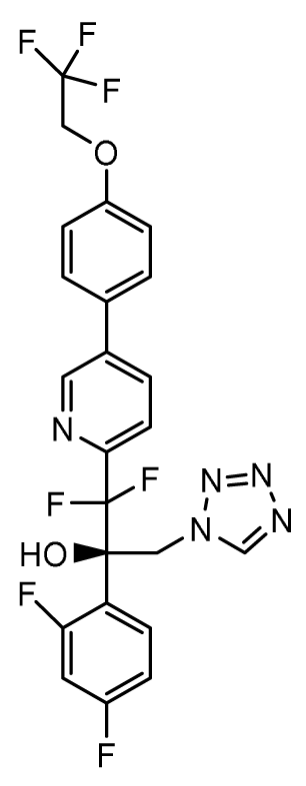 Oteseconazole 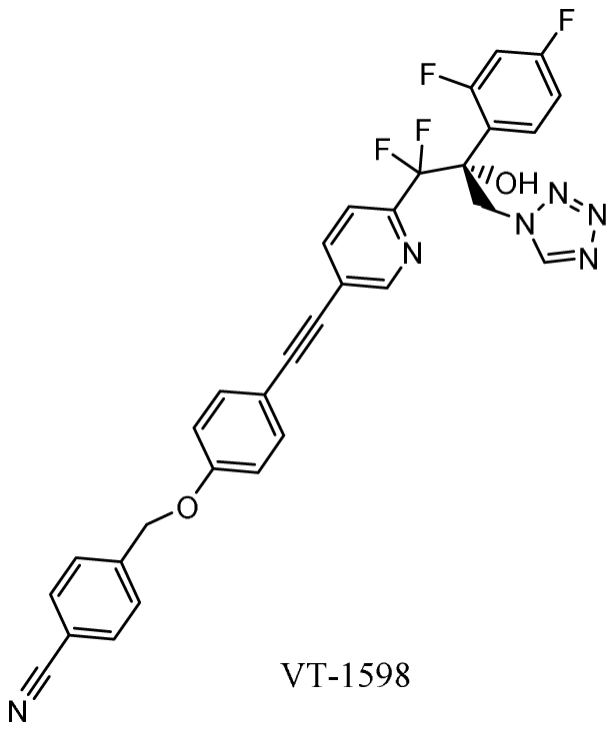	Inhibition of lanosterol 14- alpha-demethylase enzyme to disrupt ergosterol synthesis	*Candida* spp. including fluconazole- and echinocandin- resistant *C. glabrata* *Cryptococcus* spp. *Coccidioides* spp. *Histoplasma* *capsulatum* *Blastomyces dermatitidis* *Aspergillus* spp. *Rhizopus arrhizus*	Completed (Oteseconazole) • Treatment of toenail onychomycosis (NCT02267356) • Treatment of recurrent VVC (NCT02267382) • Treatment of acute vaginal candidiasis (NCT01891331) Ongoing (Oteseconazole) • Treatment of recurrent VVC (NCT02267382, NCT03562156, NCT03561701, NCT03840616) • Treatment of tinea pedis (NCT01891305)	• Fungal-specific enzyme target leads to fewer drug-drug interactions • Broad spectrum against yeasts, endemic fungi, and moulds (VT-1598)
Triazoles	PC945 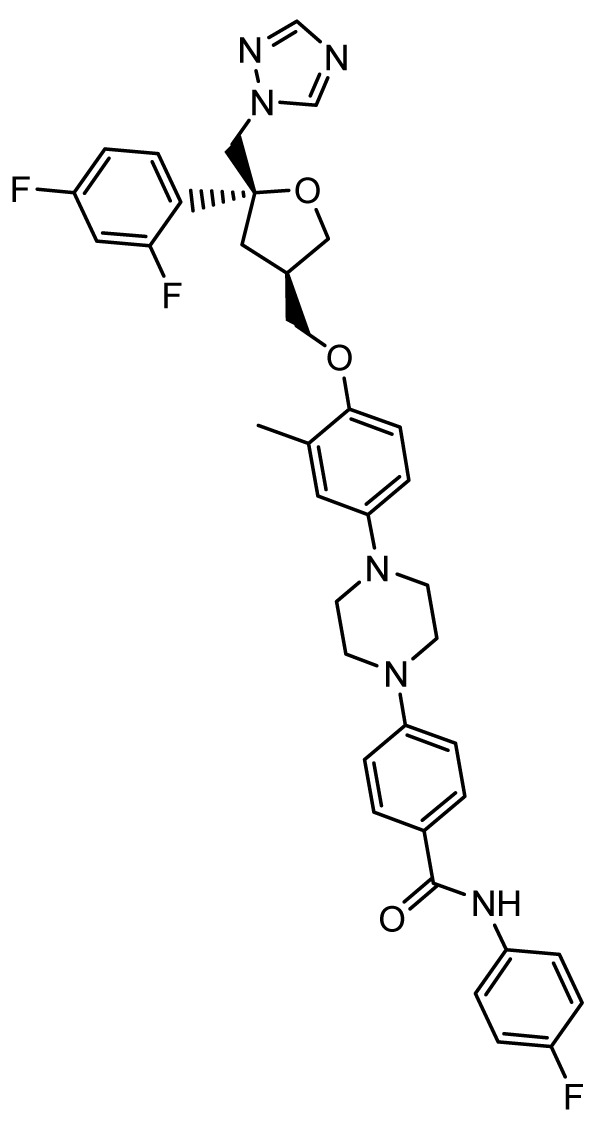	Inhibition of lanosterol 14- alpha-demethylase enzyme to disrupt ergosterol synthesis	*Candida* spp. including fluconazole-resistant *C. glabrata, C. krusei* and *C. auris* *Cryptococcus* spp. *Trichophyton rubrum* *Aspergillus fumigatus* and *A. terreus*	None	• Inhaled delivery • Activity against azole-resistant *Aspergillus* *fumigatus*
Orotomides	Olorofim (F901318) 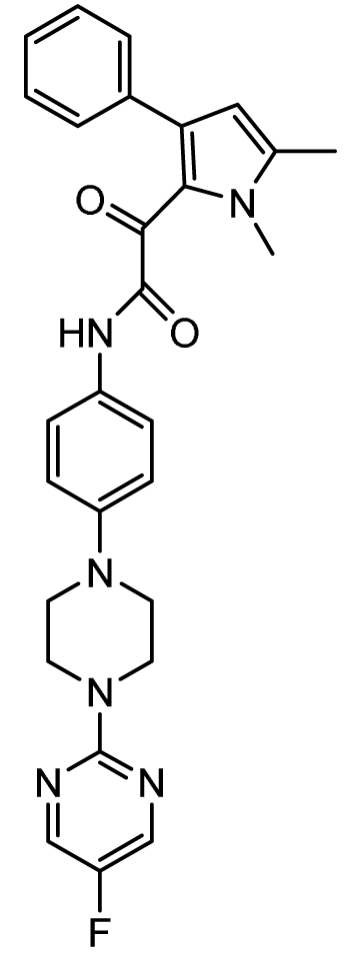	Inhibits the pyrimidine biosynthesis enzyme dihydroorotate dehydrogenase	*Aspergillus* spp. *Scedosporium* spp. *Lomentospora* *prolificans* *Fusarium* spp. *Histoplasma* *capsulatum* *Blastomyces dermatitidis* *Coccidioides* spp.	Ongoing • Treatment of IFIs due to resistant fungi (FORMULA-OLS, NCT03583164)	• Active against highly-resistant moulds

IFI, invasive fungal infection; VVC, vulvovaginal candidiasis

***Activity in vitro and in vivo*.** Fosmanogepix has broad-spectrum activity against a range of yeasts and molds. Potent *in vitro* activity is demonstrated against most *Candida* species with the exception of *Candida krusei* ^2^. Fosmanogepix also shows *in vitro* activity against fluconazole-resistant *Candida* species, including *C. auris,* as well as echinocandin-resistant *C. albicans* and *C. glabrata* with *fks* mutations ^2– 5^. Among 16 *C. auris* isolates from Europe and Asia, fosmanogepix demonstrated a minimum inhibitory concentration (MIC) required to inhibit growth of 90% of organisms (MIC _90_) value that was 8-fold lower than that of anidulafungin, the next most active agent. Highly potent *in vitro* activity was also observed in six pan-resistant *C. auris* isolates from New York (MIC range 0.008 µg/mL to 0.015 µg/mL) ^6^. Furthermore, in a neutropenic mouse model of disseminated *C. auris*, treatment with fosmanogepix led to significantly improved survival and decreased fungal burden in brain tissue as compared to anidulafungin ^5^. Fosmanogepix also was shown to have efficacy in treatment of experimental *Candida* endophthalmitis and hematogenous meningoencephalitis ^7^.

Fosmanogepix has activity against *Cryptococcus neoformans* and *C. gattii,* as well as *Coccidioides* species ^8, 9^. In mice with cryptococcal meningitis, the combination of fosmanogepix and fluconazole was observed to decrease fungal burden in a synergistic manner in brain tissue but not in lung tissue ^9^.

To determine the in vitro antifungal activity against moulds, the minimum effective concentration (MEC), rather than MIC, is determined. Amongst moulds, fosmanogepix has in vitro activity against a range of hyaline moulds including *Aspergillus spp*. ( *A. fumigatus*, *A. flavus.*, *A. niger*, *A. terreus*, *A. lentulus*, *A. ustus*), *Fusarium spp*. ( *F. solani* and *F. oxysporum* species complex), *Scedosporium spp*. ( *S. apiospermum*, *S. boydii*, *S. dehoogii*, *S. aurantiacum*), *Lomentospora prolificans*, and *Purpureocillium lilacinum* ^2, 3, 10^. For example, against *Aspergillus spp*. ( *A. fumigatus*, *Aspergillus* section *Flavi*, *Aspergillus* section *Terrei*, and *Aspergillus* section *Nigri*), fosmanogepix displayed a 50% minimal effective concentration (MEC _50_) of 0.015 *µ*g/ml; MEC _90_ of 0.03 *µ*g/ml ^3^. Fosmanogepix also exerted potent antifungal activity against *Scedosporium apiospermum* (MEC _90_ 0.12 *µ*g/ml), *Scedosporium aurantiacum* (MEC _50_ 0.06 µg/ml), and *Scedosporium (Lomentospora)* prolificans (MEC _90_ 0.12 *µ*g/ml), as well as *Fusarium solani* (MEC _90_ 0.06 *µ*g/ml) and *Fusarium oxysporum* (MEC _90_ 0.25 *µ*g/ml) ^10^. These organisms pose formidable therapeutic challenges, particularly in immunocompromised patients ^11^. In immunocompromised mouse models of invasive pulmonary aspergillosis, hematogenously disseminated fusariosis, and pulmonary scedosporiosis, fosmanogepix demonstrated improved survival and tissue clearance versus placebo; whereas, comparable outcomes were observed between mice treated with fosmanogepix and posaconazole ( *Aspergillus-*infected mice) or high dose liposomal amphotericin B ( *Fusarium-* and *Scedosporium-*infected mice) ^12, 13^. Fosmanogepix also has moderate activity against fungi in the order Mucorales (MIC ranges of 1 to 8 µg/mL) ^2^. Against *Rhizomucor* and *Rhizopus spp*., MEC ranges of 4 to >8 µg/mL are observed *in vitro* ^3^. In a mouse model of pulmonary mucormycosis with two strains of *Rhizopus arrhizus* [minimum effective concentration (MEC) values of 0.25µg/mL and 4 µg/mL], fosmanogepix lead to improved survival and reduced lung and kidney fungal burden compared to placebo and similar outcomes as compared to isavuconazole ^14^.

***Pharmacokinetics/pharmacodynamics*.** Fosmanogepix is available in oral and intravenous (IV) formulations, achieving more than 90% bioavailability in humans. In rats and monkeys administered fosmanogepix via oral or IV route, rapid and extensive absorption to most tissues including lung, brain, liver, kidney, and eye were observed. Elimination was primarily biliary (rats) and fecal (monkeys) ^15^. The 24-hour free-drug area under the concentration-time curve (AUC)/MIC ratio (fAUC _0–24_/MIC) is the PK/PD index that best correlates with efficacy in a neutropenic murine disseminated candidiasis model ( Table 2) ^16^. In phase 1 studies, plasma exposure to fosmanogepix was linear and dose proportional with a half-life of approximately 2.5 days ^17^. Fosmanogepix was well-tolerated; there was no dose-limiting toxicity, and the most common adverse event was headache ^17^. A phase 1b study of fosmanogepix safety and pharmacokinetics in patients with acute myeloid leukemia is completed, but results are not yet available ( NCT03333005).

**Table 2.** Key Pharmacodynamic Indices Associated with Therapeutic Outcome in Experimental Model Systems of Invasive Candidiasis or Invasive Aspergillosis.

Antifungal agent	Animal model	Organism	Key PD index	Reference(s)
Fosmanogepix	Murine	*C. albicans* *C. glabrata* *C. auris*	AUC/MIC	15
Ibrexafungerp	Murine	*C. albicans* *C. glabrata* *C. parapsilosis*	AUC/MIC	18, 28
Rezafungin	Murine	*C. albicans* *C. glabrata* *C. parapsilosis*	AUC/MIC	29
Olorofim	Murine	*A. flavus* *A. fumigatus*	C _min_/MIC	30, 31

PD, pharmacodynamic; AUC, area under the concentration-time curve; MIC, minimum inhibitory concentration, C _min_, minimum plasma concentration There are insufficient PK/PD data for assignment of a PD index for encochleated amphotericin B, oteseconazole, and PC945.

***Clinical development*.** Clinical development of fosmanogepix has thus far focused on its role in the treatment of infections due to *Candida* spp., *Aspergillus* spp., and rare moulds. The U.S. Food and Drug Association (FDA) has granted Fast Track, Qualified Infectious Disease Product (QIDP), and orphan drug designation to fosmanogepix for the following indications: treatment of invasive candidiasis, invasive aspergillosis, scedosporiosis, fusariosis, mucormycosis, cryptococcosis, and coccidioidomycosis. Phase 2 trials are ongoing for the treatment of IFDs caused by *Aspergillus* spp. or rare moulds ( NCT04240886), treatment of candidemia or invasive candidiasis due to *C. auris* ( NCT04148287), and treatment of candidemia in non-neutropenic patients ( NCT03604705).

### Ibrexafungerp

***Mechanism of action*.** Similar to the echinocandins, ibrexafungerp (previously MK-3118 and SCY-078; Scynexis, Jersey City, NJ, USA) disrupts fungal cell wall synthesis through inhibition of (1→3)-β-D-glucan synthase with fungicidal activity against *Candida* spp. However, ibrexafungerp is structurally distinct as a semisynthetic derivative of the naturally occurring hemiacetal triterpene glycoside enfumafungin that incorporates a pyridine triazole at position 15 of the core phenanthropyran carboxylic acid ring system and a 2-amino- 2,3,3-trimethyl-butyl ether at position 14 to enhance its antifungal potency and pharmacokinetic properties; thus, representing the first compound in the novel class of triterpenoid antifungals ^18^. As compared to echinocandins, ibrexafungerp has distinct advantages of oral bioavailability, broad activity against pan-resistant *C. auris,* and maintaining activity against most echinocandin-resistant *Candida* spp. 

***Activity in vitro and in vivo*.** Ibrexafungerp exhibits potent fungicidal activity against *Candida* species, including *C. glabrata* and multiple clades of *C. auris* ^19– 21^. The in vitro activity of ibrexafungerp against *C. parapsilosis*, which is known to have elevated echinocandin MICs, is comparable to or improved from that of the echinocandins ^22^. The modal MIC of ibrexafungerp against C. parapsilosis is also similar to the modal MIC observed against other common *Candida* species causing clinical disease, *C. albicans*, *C. glabrata*, *C. tropicales*, and *C. krusei* ^23^. Notably, ibrexafungerp retains *in vitro* activity against most echinocandin-resistant *C. glabrata* with *fks* mutations (MIC mode, MIC _50_, and MIC _90_ of 0.25µg/mL, 0.25µg/mL, and 1.0µg/mL, respectively) ^22^. Amongst *C. auris* isolates with echinocandin resistance or pan-antifungal resistance, ibrexafungerp demonstrates MIC ranges from 0.25µg/mL to 1µg/mL and 0.12µg/mL to 1µg/mL, respectively ^20, 24, 25^. In addition, *C. auris* biofilms treated with ibrexafungerp show reduced metabolic activity and thickness as compared to untreated control biofilms ^26^.

Ibrexafungerp has fungistatic activity against *Aspergillus* species (MEC range <0.06µg/mL to 4µg/mL) ^27^. The combination of ibrexafungerp with voriconazole, amphotericin B, or isavuconazole demonstrates *in vitro* synergy against wild-type (WT) *Aspergillus* species but not against azole-resistant strains ^27^. Little *in vitro* activity is observed with ibrexafungerp against the Mucorales and non- *Aspergillus* hyaline moulds ( *Fusarium* spp, *Scopulariopsis* spp, *Lomentospora prolificans*) with the exception of *Paecilomyces variotii* (MEC <0.02µg/mL to 0.03µg/mL) ^32^. However, synergistic interaction between ibrexafungerp and isavuconazole is observed *in vitro* against *Cunninghamella bertholletiae*, *S. apiospermum*, *F. solani* and *F. oxysporum*; whereas, indifference or antagonism are observed with *Mucor circinelloides* and *Rhizopus* species, respectively ^33^.

In a murine invasive candidiasis model with WT and echinocandin-resistant (ER) *C. glabrata*, ibrexafungerp significantly reduced kidney fungal burden in both groups as compared to placebo. In contrast, caspofungin administered by intraperitoneal injection reduced fungal burden in the WT group but not the ER group ^34^. Reduced tissue fungal burden and improved survival with ibrexafungerp versus control also were observed in immunocompromised mice with disseminated *C. auris* ^35^.

In a murine model of disseminated aspergillosis, treatment with ibrexafungerp led to significant reduction in *Aspergillus* kidney burden and serum galactomannan (GM) levels and improved survival as compared to control ^36^. This *in vivo* activity of ibrexafungerp was observed in both wild type and azole-resistant isolates of *A. fumigatus*.

The combination of ibrexafungerp and isavuconazole also demonstrates synergy in a neutropenic rabbit model of experimental invasive pulmonary aspergillosis. As compared to isavuconazole alone, mice treated with ibrexafungerp and isavuconazole had significantly improved survival, decreased pulmonary infarct scores, and diminished serum GM levels ^37^.

Ibrexafungerp is also efficacious in a murine model of *Pneumocystis murina* pneumonia, in which reductions in asci burden and improvements in survival were similar to those of trimethoprim-sulfamethoxazole and significantly better than in untreated controls ^38^.

***Pharmacokinetics/pharmacodynamics*.** Ibrexafungerp is orally bioavailable and highly protein bound (~99.6%) in humans. The maximum plasma concentration (C _max_) and area under the concentration-time curve (AUC) increase approximately 20% with high fat meals ^39^. It has a large volume of distribution in mice, rats, and dogs. Concentration in multiple tissues including liver, spleen, lungs, bone marrow, kidney, and skin exceeds that of plasma. However, there is low distribution to central nervous system (CNS) tissue ^40^. In rats, approximately 90% of drug is eliminated in feces and bile, and 1.5% eliminated in urine ^40^. The PK/PD index that best correlates with efficacy in a murine models of disseminated candidiasis is AUC/MIC ( Table 2) ^18, 41^. Ibrexafungerp is a substrate of CYP3A and P-glycoprotein, though it neither induces or nor inhibits CYP3A. When ibrexafungerp and tacrolimus are co-administered, there is a 1.4-fold increase in AUC and no change in tacrolimus C _max_^42^. Thus, initial tacrolimus dose adjustment is not needed when co-administered with ibrexafungerp.

***Clinical development*.** Ibrexafungerp will likely play an important role in management of invasive candidiasis due to WT and resistant *Candida* species and invasive aspergillosis; the drug has received QIDP and orphan drug designations for both indications.

In a phase 2 open-label, randomized study, 27 patients with invasive candidiasis were randomized to receive step-down therapy to one of three treatment arms: two dosing regimens of ibrexafungerp (1000mg loading dose followed by 500mg daily or 1250mg loading dose followed by 750mg daily) or standard of care (SOC) following initial echinocandin therapy. Similar rates of adverse events were observed across study arms; study-drug related treatment-emergent adverse events were reported in two patients (vomiting and diarrhea) and did not require drug discontinuation. There was no difference in favorable global response rates (clinical and microbiologic): 86%, 71%, and 71% in the ibrexafungerp 750mg, ibrexafungerp 500mg, and SOC arms, respectively, although the study was not powered to detect statistical superiority ^28^.

A phase 3 open-label, single arm study of ibrexafungerp in patients with refractory or intolerant fungal diseases is ongoing (FURI; NCT03059992). An interim analysis was performed in 20 patients with proven or probable invasive candidiasis (N=11) or severe mucocutaneous candidiasis (N=9). Eleven (55%) patients achieved a complete or partial response and 6 (30%) had stable disease. The most common treatment-related adverse events were gastrointestinal ^43^. Target enrollment is 200 patients, and the estimated study completion date is December 2021.

Ibrexafungerp has also been studied for the treatment of vulvovaginal candidiasis (VVC). Day 10 and day 25 clinical cure and mycological eradication rates were similar or improved with ibrexafungerp 300mg twice daily × 2 doses compared to fluconazole 150mg × 1 dose. Diarrhea was the most common adverse event in the ibrexafungerp arm, observed in 10% of subjects ^44^. An new drug application has since been submitted for treatment of VVC.

Other ongoing clinical trials include a multicenter, randomized, double-blind study to evaluate the efficacy and safety of ibrexafungerp and voriconazole in patients with invasive pulmonary aspergillosis ( NCT03672292). Ibrexafungerp is also in open-label clinical trials in India and the United States for treatment of *Candida auris* infection (CARES; NCT03363841). Thus far, outcomes of two patients enrolled in the CARES Study have been reported; both had *C. auris* bloodstream infections and were successfully treated with ibrexafungerp ^45^.

Combination antifungal therapy with a cell wall active agent and an antifungal triazole is a potentially important strategy in treatment of invasive aspergillosis [33]. Ibrexafungerp may develop a key role in combination antifungal therapy with an antifungal triazole in treatment of invasive aspergillosis. Simultaneous administration of an orally administered triazole and ibrexafungerp may allow patients to receive the potential therapeutic benefit of combination therapy in treatment of invasive pulmonary aspergillosis on an ambulatory basis.

### Rezafungin

***Mechanism of action*.** Rezafungin (formerly SP3025 and CD101; Cidara Therapeutics, San Diego, CA, USA) is a novel agent in the echinocandin antifungal drug class that inhibits (1→3)-β-D-glucan synthesis. Rezafungin is a structural analogue of anidulafungin but it is differentiated by a choline moiety at the C5 ornithine position, conferring increased stability and solubility ^46^. Due to its long half-life, rezafungin has the advantage of once weekly dosing as compared to other drugs within the echinocandin class that require daily dosing.

***Activity in vitro and in vivo*.** Rezafungin has potent *in vitro* activity that mirrors that of other echinocandins against WT and azole-resistant *Candida* species, as well as WT and azole-resistant *Aspergillus* species ^47– 49^. Similar to other echinocandins, rezafungin has higher MICs (MIC _50_ 1 µg/mL/MIC _90_ 2 µg/mL) against *C. parapsilosis* compared to other common *Candida species*.

In immunocompromised mouse models of *C. albicans* and *A. fumigatus* infection, decreased fungal tissue burden and improved 10-day survival, respectively, were observed with rezafungin as compared to controls ^50^. Rezafungin also had activity in a mouse model of disseminated *C. auris*, leading to decreased fungal tissue burden as compared to amphotericin B and control ^51^. Furthermore, rezafungin was efficacious as prophylaxis against *Pneumocystis* in a mouse model, supporting its potential for development for prevention of *Pneumocystis* pneumonia in immunocompromised hosts ^52^.

***Pharmacokinetics/pharmacodynamics*.** Similar to other echinocandin drugs, rezafungin demonstrates a concentration-dependent pattern of fungicidal activity. Therefore, a front-loaded dosing regimen conferring higher plasma drug exposure may theoretically enhance pathogen killing and raise the barrier to drug resistance ^53^. In animal models, the AUC/MIC correlates best with therapeutic outcome ^54^ ( Table 2). In phase 1 ascending dose studies evaluating single doses up to 400mg and multiple doses up to 400mg once weekly for 3 weeks in healthy adults, rezafungin demonstrated dose-proportional plasma exposures, long half-life (approximately 80 hours after the first dose and 152 hours after the third dose), and minimal renal excretion ^55^. The C _max_ ranged from ~5 µg/mL with the 100mg dose to ~22 to 30 µg/mL with the 400mg dose. Overall, rezafungin was well tolerated. There were no serious adverse events; most adverse events were mild and gastrointestinal (constipation and nausea). Mild infusion reactions characterized by nausea, flushing, and chest discomfort were also observed, most often with the third dose of 400mg of rezafungin. These reactions resolved within minutes without drug interruption or discontinuation. 

***Clinical development*.** Rezafungin has received U.S. FDA QIDP and Fast Track designations for prevention of invasive fungal infections as well as QIDP, Fast Track, and orphan drug designations for treatment of invasive candidiasis. 

A phase 2 multicenter, randomized, double-blinded trial in 207 adult patients with candidemia and/or invasive candidiasis compared the efficacy and safety of treatment with rezafungin versus caspofungin with fluconazole stepdown once clinically stable (STRIVE; NCT02734862) ^56^. Patients were randomized to one of three treatment arms: rezafungin 400mg once weekly, rezafungin 400mg on week 1, then 200mg weekly, and caspofungin 70mg loading dose followed by 50mg daily for ≤4 weeks. The primary endpoint was overall cure, defined as resolution of signs of candidemia or invasive candidiasis and mycological eradication at day 14. The study was not designed for statistical comparison of the efficacy assessment, but overall cure rates and 30-day mortality, respectively, were similar across groups: rezafungin 400mg weekly (60.5% and 15.8%), rezafungin 400mg/200mg weekly (76.1% and 4.4%), and caspofungin (67.2% and 13.1%). In patients with candidemia, blood cultures cleared in 19.5 and 22.8 hours in the rezafungin and caspofungin groups, respectively. Rezafungin was also well-tolerated. The most common adverse events – hypokalemia, diarrhea, and vomiting – were observed in similar proportions of patients in the rezafungin and caspofungin groups. Study drug-related serious adverse events occurred in one patient in each rezafungin group and two patients in the caspofungin group. 

Based on the promising results of STRIVE, a phase 3 clinical trial of rezafungin versus caspofungin for treatment of candidemia and invasive candidiasis is ongoing (ReSTORE; NCT03667690). Another ongoing phase 3 trial compares rezafungin to standard of care for prevention of IFD due to *Candida* spp., *Aspergillus* spp., and *Pneumocystis* in patients undergoing allogeneic hematopoietic cell transplantation (ReSPECT; NCT04368559). The primary outcome is fungal-free survival at Day 90. In both phase 3 trials, rezafungin is dosed 400mg for the first week followed by 200mg once weekly.

## Agents targeting the cell membrane

### Encochleated Amphotericin B (MAT2203)

***Mechanism of action and pharmacology*.** Amphotericin B (AmB), a polyene antifungal agent, disrupts fungal cell wall synthesis by binding to ergosterol to form pores that allow leakage of intracellular contents, resulting in potent fungicidal activity against a wide range of yeasts and moulds. However, AmB and its lipid formulations are only available via intravenous injection due to low solubility, a tendency to self-aggregate in aqueous media, and low permeability ^29^. Encochleated AmB (CAmB; Matinas BioPharma, Bedminster, NJ, USA) is a novel formulation that allows for oral administration with reduced toxicity. Cochleates form a multilayered structure composed of a negatively charged lipid (phosphatidylserine) and a divalent cation (calcium). This structure protects AmB from degradation within the gastrointestinal tract ^57^. AmB is released to the fungus only when the cochleates interact with the target cells and subsequently destabilize in the setting of low intracellular calcium concentration.

***Activity in vitro and in vivo*.** Comparable *in vitro* activity against *Candida* spp. and *Aspergillus* spp. are observed with CAmB and deoxycholate AmB ^58, 59^. CAmB has been successfully administered in immunocompromised mouse models of disseminated *C. albicans* infection and disseminated aspergillosis. In both studies, oral CAmB and intraperitoneal deoxycholate amphotericin B demonstrated similar improvement in survival and reduction in tissue fungal burden as compared to untreated control animals ^58, 60^. Furthermore, CAmB was evaluated in a mouse model of cryptococcal meningoencephalitis where CAmB plus flucytosine had similar efficacy to parenteral AmB plus flucytosine and demonstrated potent activity ^61^.

***Pharmacokinetics/pharmacodynamics*.** A single dose of CAmB demonstrates extensive tissue distribution and penetration into target tissues in animal models ^62^. In a phase 1 study in healthy adults evaluating escalating doses of 200, 400, and 800mg, CAmB was well tolerated at doses of 200mg and 400mg. The most common adverse events were gastrointestinal, occurring in 6%, 38%, and 56% of patients in the 200mg, 400mg, and 800mg groups, respectively. There were no serious adverse events or renal toxicity observed. Dose-dependent increases in C _max_ and AUC were observed, comparable to those of animal toxicity studies ^63^.

***Clinical development*.** A phase 2a single-arm study of CAmB for refractory mucocutaneous candidiasis is ongoing ( NCT02629419). Preliminary results indicate that all enrolled patients met the primary endpoint of ≥ 50% improvement in clinical signs and symptoms. CAmB was well tolerated at 400mg and 800mg with no observed renal or hepatic toxicity. In a phase 2 study of CAmB 200mg and 400mg and fluconazole 150mg for VVC in 137 patients, lower rates of clinical cure and more adverse events were observed with CAmB 200mg and 400mg as compared to fluconazole ( NCT02971007). There were no serious adverse events ^64^. Phase 1 and 2 studies of CAmB for treatment of cryptococcal meningitis in HIV-infected patients in Uganda are ongoing (EnACT; NCT04031833). CAmB has FDA-granted Fast Track, QIDP, and orphan drug designations for treatment of invasive candidiasis and aspergillosis, prevention of IFDs in patients on immunosuppressive therapy, and treatment of cryptococcosis.

### Oteseconazole (VT-1161), VT-1598, VT-1129

***Mechanism of action*.** Second-generation triazole antifungal agents, such as voriconazole, are highly effective against a range of yeasts and moulds; however, they are associated with significant drug-drug interactions due to off-target inhibition of human cytochrome P450 enzymes. Oteseconazole (VT-1161), VT-1598, and VT-1129 (Mycovia Pharmaceuticals, Inc., Durham, NC, USA) are next-generation azoles in which selective inhibition of the fungal enzyme CYP51 is more readily achieved by replacing the 1-(1,2,4-triazole) metal-binding group with a tetrazole ^65^.

***Activity in vitro and in vivo*.** Oteseconazole, VT-1598, and VT-1129 have potent *in vitro* activity against *Cryptococcus* spp. and *Candida* spp. including *C. krusei* and fluconazole- and echinocandin-resistant *C. glabrata* ^66, 67^. VT-1598 has the broadest spectrum, which includes *C. auris*, moulds ( *Aspergillus* spp. and *Rhizopus* spp.) and endemic dimorphic fungi ( *Histoplasma capsulatum*, *Blastomyces dermatitidis*, *Coccidioides posadasii,* and *C. immitis*) ^68– 70^. In murine models of CNS coccidioidomycosis, VT-1598 treatment leads to improved survival and reduced fungal burden in brain tissue as compared to fluconazole. Oteseconazole has similarly demonstrated efficacy in murine models of pulmonary and CNS coccidioidomycosis as well as disseminated mucormycosis due to *Rhizopus arrhizus* var. *arrhizus* ^71, 72^.

***Clinical development*.** The FDA has granted QIDP, fast track, and orphan drug designation to VT-1598 for the treatment of coccidioidomycosis (Valley fever) ^73^. VT-1598 is in phase 1 studies ( NCT04208321). Oteseconazole is in phase 3 clinical trials for treatment of recurrent vaginal candidiasis ( NCT02267382, NCT03562156, NCT03561701) after demonstrating safety and efficacy in a phase 2 study and has FDA QIDP and Fast-Track designations for this indication ^74^. A phase 2 trial for toenail onychomycosis demonstrated higher week 48 cure rates with oteseconazole (32 to 42%) versus placebo (0%) ( NCT02267356) ^75^. In these completed trials, oteseconazole was well-tolerated with no evidence of hepatotoxicity or QT prolongation.

### PC945

***Mechanism of action*.** As compared to systemic therapy, aerosolized delivery of antifungal agents to the lung results in higher concentrations in epithelial lining fluid and bronchoalveolar lavage fluid; however, for successful activity, drug levels must be sustained in lung tissues with minimal systemic absorption ^76^. PC945 (Pulmocide, London, United Kingdom) is a novel triazole antifungal agent that is being developed specifically for inhaled administration for treatment and prevention of invasive fungal infections of the sinopulmonary tract. The structure of PC945 is similar to but distinct from that of posaconazole. The structures are similar in having 2,4-difluorophenyl and 1H-1,2,4-triazole substitutions on the asymmetric carbon atom. However, PC945 differs structurally in having a central oxolane ring (in place of the dioxalane ring) and a long hydrophobic 3-ylmethoxy-3-methylphenyl[piperazin-1-yl]-N-(4-fluorophenyl)benzamide substitution. This hydrophobic moiety likely contributes to the sustained intrapulmonary concentrations of PC945.

***Activity in vitro and in vivo*.** PC945 has *in vitro* activity against azole-susceptible *A. fumigatus* [median MIC 0.031µg/mL (IQR 0.02 – 0.031µg/mL)] and most azole-resistant *A. fumigatus* ^77^. Activity against *A. terreus* is comparable to posaconazole and more potent than that of voriconazole; however, PC945 has poor *in vitro* activity against *A. flavus* and *A. niger*. PC945 lacks activity against most Mucorales; although a MIC 2µg/mL was observed for *Rhizopus oryzae* ^77^. Against *Candida albicans* (both azole-susceptible and azole-resistant strains), *C. glabrata*, and *C. krusei*, PC945 is generally more active than voriconazole and shares equal potency with posaconazole ^77^. Using a global collection of 50 clinical *Candida auris* isolates, PC945 had more potent *in vitro* activity than posaconazole, voriconazole, and fluconazole [PC945 GM MIC (MIC _50_, MIC _90_): 0.14µg/mL (0.13, 1µg/mL)] ^78^.

An *in vitro* model of the human alveolus has been developed to better understand the pathogenesis of invasive pulmonary aspergillosis and the relationship between the kinetics of GM and outcomes of antifungal therapy ^79^. Using this model, combination therapy with apical PC945 and basolateral posaconazole or voriconazole for azole-susceptible and azole-resistant *A. fumigatus* demonstrated synergistic activity as compared to either agent alone ^80^.

The therapeutic potential of intranasal PC945 has been investigated in transiently neutropenic mice with invasive pulmonary aspergillosis. Intranasal PC945 leads to reduced concentrations of GM in bronchoalveolar lavage fluid (BALF) and serum and improved survival as compared to controls, and reduced GM concentration and similar survival as compared to intranasal posaconazole ^77, 81^. Combination therapy with intranasal PC945 and oral posaconazole was also evaluated in immunocompromised neutropenic mice with azole-susceptible *A. fumigatus* infection. Suboptimal dosages of PC945 and posaconazole were administered simultaneously, (i.e., doses at which either agent alone led to zero survival at Day 7), and Day 7 survival improved to 83% ^80^. As a potential prophylactic agent, PC945 was administered in the same *A. fumigatus*-infected mouse model from days -7 to +3 and days -1 to +3. Extended prophylaxis (days -7 to +3) yielded greater inhibition of fungal load in lung tissue and GM concentrations in BALF and serum as compared to shorter duration, suggesting that the antifungal effects of PC945 accumulated in the lung upon repeat dosing ^81^.

***Pharmacokinetics/pharmacodynamics*.** Using the human alveolus, topical PC945 demonstrates sustained residency and antifungal activity in epithelial cells ^80^.

***Clinical development*.** A phase 1 trial of PC945 in 29 healthy subjects and patients with mild asthma is completed; results are not yet available on clinicaltrials.gov ( NCT02715570). A phase 3 study of PC945 for adults, who have limited or no alternative treatment options, for the treatment of invasive pulmonary aspergillosis as part of a combined antifungal regimen is planned to start in 2021. 

In a report of two lung transplant recipients with bronchial anastomotic masses due to *A. fumigatus*, PC945, administered in combination with systemic antifungal agents, was well-tolerated, and clinical resolution of infection was observed ^82^.

## Agents targeting nucleic acid metabolism

### Olorofim

***Mechanism of action*.** Olorofim (previously F910318, discovered by F2G Ltd, Australia), a member of the novel antifungal class, orotomides, is in an inhibitor of the pyrimidine biosynthesis fungal enzyme dihydroorotate dehydrogenase. Interruption of pyrimidine synthesis impairs nucleic acid production and leads to the arrest of hyphal extension ^83^.

***Activity in vitro and in vivo*.** Olorofim is unique among existing antifungal agents in that it has no activity against *Candida* species. Rather, olorofim has potent activity *in vitro* against WT and azole-resistant *Aspergillus* spp., some other highly resistant hyaline moulds, and *Coccidioides* spp. ^84^ Amongst 133 azole-resistant *A. fumigatus* isolates due to TR34/L98H,TR46/Y121F/T289A, *cyp51A*-associated point mutations, or unknown resistance mechanisms, MIC range was 0.031µg/mL to 0.125µg/mL, 0.062µg/mL to 0.25µg/mL, and 0.01µg/mL to 0.125µg/mL, respectively ^85^. Several studies have also shown excellent activity *in vitro* against *Scedosporium* species (MIC _50_/MIC _90_ 0.06/0.25µg/mL) and *L. prolificans* (MIC _50_/MIC _90_ 0.12/0.2µg/mL) including biofilm formation by the latter ^86– 88^. The geometric mean MICs of olorofim were significantly lower for all *Scedosporium* species and *L. prolificans* compared with those of voriconazole, posaconazole, amphotericin B, and caspofungin ^86^. Less *in vitro* data are available for *Fusarium* species, but susceptibility appears to be species-specific, with lower MICs observed for *F. proliferatum* than *F. solani* species complex and *F. dimerum* ^89^. Olorofim has no activity against the Mucorales or the dematiaceous pathogen *Exophiala dermatitidis* ^83, 90^.

In murine models of profound neutropenia and chronic granulomatous disease with disseminated and pulmonary aspergillosis, respectively, intraperitoneal administration of olorofim lead to significantly reduced serum GM levels and organ fungal DNA burden and improved survival as compared to controls ^30^. In a murine model of acute sinopulmonary aspergillosis due to *A. flavus*, olorofim had comparable antifungal activity to posaconazole for the outcomes of decline in GM, histologic clearance of lung tissue, and survival ^31^.

***Pharmacokinetics/pharmacodynamics*.** Olorofim is available in oral and IV formulations and demonstrates time-dependent antifungal activity ^31, 91^. Olorofim initially has a fungistatic effect on *Aspergillus* isolates but prolonged exposure is fungicidal ^92^. Pharmacokinetic studies in mice have identified good distribution of olorofim to tissues including the kidney, liver, and lung, with lower levels of detection in the brain ^83^. Olorofim exhibits time-dependent antifungal activity; the PK/PD index minimum inhibitory concentration (C _min_)/MIC strongly correlates with treatment outcome in murine models of invasive aspergillosis ^30, 31^ ( Table 2). In a phase 1 study of multiple doses of an immediate-release tablet (360mg daily for 10 days), steady state was reached within three days of dosing, and once attained, mean plasma trough levels were 1 to 2µg/mL and exceeded 0.7µg/mL in all subjects. There was evidence of enterohepatic recirculation. Olorofim was well-tolerated in all eight subjects with no serious adverse events and no subject withdrawn due to an AE. Drug-related adverse events included increased ALT (N=2), nausea and diarrhea (N=1), and dizziness (N=1) ^93^. Olorofim is a weak inhibitor of CYP3A4 ^94^.

***Clinical development*.** Olorofim received designation from the U.S. FDA as a breakthrough therapy in 2019 and as an orphan drug in 2020. The European Medicines Agency Committee for Orphan Medicinal Products also granted orphan drug status to olorofim for the treatment of invasive aspergillosis and scedosporiosis in March 2019. A phase 2 clinical trial of olorofim for the treatment of IFDs due to resistant fungi including azole-resistant aspergillosis, scedosporiosis, and lomentosporiosis is ongoing (FORMULA-OLS; NCT03583164) as is a phase 1 drug-drug interaction study with itraconazole and rifampicin ( NCT04171739).

## Conclusion

Despite significant advances in prevention, diagnosis, and management of IFDs over the past several decades, IFDs remain a formidable threat to immunocompromised hosts. In addition to strategies to augment host response and reduce immunosuppression, novel therapeutics with potent fungicidal activity and low toxicity are urgently needed. We review investigational drugs in clinical phases of development, including three agents within three novel antifungal classes targeting the fungal cell wall and nucleic acid metabolism, fosmanogepix, olorofim, and ibrexafungerp. Fosmanogepix and olorofim are unique in their potent activity against highly-resistant molds for which we have few, if any, effective agents in our current antifungal armamentarium. Ibrexafungerp has selective advantage as an oral fungicidal therapy for *Candida* species including echinocandin-resistant *C. glabrata* and *C. auris*. Four other investigational agents within existing antifungal drug classes, rezafungin, CAmB, oteseconazole, and PC945, demonstrate enhanced pharmacokinetic and pharmacodynamic properties that importantly afford improved safety and tolerability profiles. Rezafungin, in particular, with its once weekly dosing, may facilitate outpatient management of patients needing an echinocandin for treatment or prophylaxis. Overall, we are optimistic that the current antifungal pipeline will expand our ability to provide safe and effective treatments to patients suffering from IFDs.

## Data availability

No data is associated with this article.
