# Supplementary material for: Novel antifungal agents in clinical trials
Source: F1000Res. 2022 Jan 12;10:507. Originally published 2021 Jun 28. [Version 2] doi: 10.12688/f1000research.28327.2 (PMC8787557; doi:10.12688/f1000research.28327.2)
Supplement: Peer review history

# Peer review: Reviewer response for version 2

**DOI:** 10.5256/f1000research.119851.r119566

**Published:** 2022-01-24

**Authors:** Shahram Mahmoudi

**Affiliations:** 1 Department of Parasitology and Mycology, School of Medicine, Iran University of Medical Sciences, Tehran, Iran

I have no further comments to make.

Is the review written in accessible language?

Yes

Are all factual statements correct and adequately supported by citations?

Yes

Are the conclusions drawn appropriate in the context of the current research literature?

No

Is the topic of the review discussed comprehensively in the context of the current literature?

Yes

Reviewer Expertise:

Medical Mycology, Antifungal Drugs

I confirm that I have read this submission and believe that I have an appropriate level of expertise to confirm that it is of an acceptable scientific standard.

# Peer review: Reviewer response for version 1

**DOI:** 10.5256/f1000research.31334.r99295

**Published:** 2021-12-02

**Authors:** Shahram Mahmoudi

**Affiliations:** 1 Department of Parasitology and Mycology, School of Medicine, Iran University of Medical Sciences, Tehran, Iran

The study by Jacobs et al. summarizes the antifungal drugs in clinical trials. The corresponding author is experienced, the study is well written, and when looked alone, it seems perfect.

 However, I think the following points need to be considered by the authors:

- Please be consistent in the use of units. In some cases, the unites are linked to the numbers (e.g. 35mg/L), and in the rest are separated by a space (e.g. 35 mg/L). Please add one space between numbers and units throughout the manuscript.
- In my opinion, conclusion needs to be changed as in its current form it looks like the aim of study and not a conclusion.

Is the review written in accessible language?

Yes

Are all factual statements correct and adequately supported by citations?

Yes

Are the conclusions drawn appropriate in the context of the current research literature?

No

Is the topic of the review discussed comprehensively in the context of the current literature?

Yes

Reviewer Expertise:

Medical Mycology, Antifungal Drugs

I confirm that I have read this submission and believe that I have an appropriate level of expertise to confirm that it is of an acceptable scientific standard, however I have significant reservations, as outlined above.

# Response

**Published:** 2021-12-27

**Authors:** Samantha Jacobs

**Affiliations:** Icahn School of Medicine at Mount Sinai, USA

**Response to Reviewer #2 Comments**

**12.27.2021**

 The study by Jacobs et al. summarizes the antifungal drugs in clinical trials. The corresponding author is experienced, the study is well written, and when looked alone, it seems perfect.

 However, I think the following points need to be considered by the authors:

- Please be consistent in the use of units. In some cases, the unites are linked to the numbers (e.g. 35mg/L), and in the rest are separated by a space (e.g. 35 mg/L). Please add one space between numbers and units throughout the manuscript.

**Author response:** We have added one space between numbers and units throughout the manuscript.

- In my opinion, conclusion needs to be changed as in its current form it looks like the aim of study and not a conclusion

**Author response**:  We have modified the conclusion as suggested by the reviewer to summarize and highlight the advantages of the most promising investigational agents discussed in the manuscript.

# Peer review: Reviewer response for version 1

**DOI:** 10.5256/f1000research.31334.r89343

**Published:** 2021-07-23

**Authors:** Charalampos Antachopoulos

**Affiliations:** 1 Third Department of Pediatrics, Faculty of Medicine, School of Health Sciences, Hippokration General Hospital, Aristotle University, Thessaloniki, Greece

This is a well-written and comprehensive paper summarizing recent data on novel antifungal agents in clinical development. Table 1 is very helpful. I have only few minor comments:

1. Abstract: consider change “we examine” to: “we review / present / discuss”.
2. Fosmanogepix: please provide more specific *in vitro *data on species (rather than genera) susceptibility for *Aspergillus *spp, *Fusarium* spp and *Scedosporium* spp.
3. *In vitro* antifungal activity of fosmanogepix: it should be mentioned that MEC rather than MIC is determined for assessment of activity against molds.
4. “Fosmanogepix also has activity against fungi in the order Mucorales (MIC ranges of 1 to 8 µg/mL)”: please provide reference for this statement; is the term MIC correct or should it be replaced by MEC?
5.  “Ibrexafungerp has fungistatic activity against *Aspergillus* species (MIC range <0.06 µg/mL to 32 µg/mL…)”: in the reference cited by the authors (ref. 25, Ghannoum et al, AAC 2018), ibrexafungerp *in vitro *activity against *Aspergillus* had been determined using both MEC and MIC; the MIC values tended to be 8-fold higher than correspondent MEC values. Please modify the text accordingly.
6. Are there data on the *in vitro* activity of ibrexafungerp and rezafungin against *Candida parapsilosis* in comparison with other *Candida* species?
7. What is the published evidence for PK/PD indices associated with therapeutic outcome for each one of the novel antifungal agents presented in this paper?
8. Conclusion: the content of the conclusion paragraph would fit more for an introduction rather than a wrap-up of the main findings. I suggest to significantly modify this section, in order to briefly mention the novel agents in clinical development and their selective advantages compared to our current antifungal armamentarium.

Is the review written in accessible language?

Yes

Are all factual statements correct and adequately supported by citations?

Yes

Are the conclusions drawn appropriate in the context of the current research literature?

Yes

Is the topic of the review discussed comprehensively in the context of the current literature?

Yes

Reviewer Expertise:

1. Antifungal agents (in vitro activity against planktonic fungal cells or biofilms); 2. Host immune responses against fungal infections; 3. Pharmacokinetics and safety of novel antimicrobial agents in pediatric patients

I confirm that I have read this submission and believe that I have an appropriate level of expertise to confirm that it is of an acceptable scientific standard.

# Response

**Published:** 2021-12-27

**Authors:** Samantha Jacobs

**Affiliations:** Icahn School of Medicine at Mount Sinai, USA

**Response to Reviewer #1 Comments**

**12.27.2021**

 This is a well-written and comprehensive paper summarizing recent data on novel antifungal agents in clinical development. Table 1 is very helpful. I have only few minor comments:

 1. Abstract: consider change “we examine” to: “we review / present / discuss”.

**Author response**: We have changed the wording to “We discuss…”

 2. Fosmanogepix: please provide more specific *in vitro *data on species (rather than genera) susceptibility for *Aspergillus *spp, *Fusarium* spp and *Scedosporium* spp.

**Author response**: We have specified the *in vitro* activity of fosmanogepix to the species level including representative MEC values.

*3. In vitro* antifungal activity of fosmanogepix: it should be mentioned that MEC rather than MIC is determined for assessment of activity against molds.

**Author response**: We have added a line noting this distinction.

 4. “Fosmanogepix also has activity against fungi in the order Mucorales (MIC ranges of 1 to 8 µg/mL)”: please provide reference for this statement; is the term MIC correct or should it be replaced by MEC? **Author response**: We have added the reference for this statement (reference #2).  Indeed, the manuscript, published in 2011, used MIC rather the MEC determination for activity of fosmanogepix against *R. oryzae* and *R. microsporus*. We have also added another reference that reports MEC values (reference #3).

 5. “Ibrexafungerp has fungistatic activity against *Aspergillus* species (MIC range <0.06 µg/mL to 32 µg/mL…)”: in the reference cited by the authors (ref. 25, Ghannoum et al, AAC 2018), ibrexafungerp *in vitro *activity against *Aspergillus* had been determined using both MEC and MIC; the MIC values tended to be 8-fold higher than correspondent MEC values. Please modify the text accordingly.

**Author response**: Thank you for your comment. We have modified the text to report the MEC rather than MIC determinations for ibrexafungerp against *Aspergillus* species.

 6. Are there data on the *in vitro* activity of ibrexafungerp and rezafungin against *Candida parapsilosis* in comparison with other *Candida* species?

**Author response:** Thank you for your question.  The *in vitro* activity of ibrexafungerp against *C. parapsilosis* is comparable or more potent than that of echinocandins. The MICs against *C. parapsilosis* are generally similar to those of other *Candida* species. For rezafungin, the *in vitro* activity against *C. parapsilosis *is less potent than against other common *Candida* species causing disease. These data are added to the text.

 7. What is the published evidence for PK/PD indices associated with therapeutic outcome for each one of the novel antifungal agents presented in this paper?

**Author response**:  For fosmanogepix, ibrexafungerp, rezafungin, and olorofim, we have included the key PK/PD indices associated with therapeutic outcome in experimental animal models as there are insufficient clinical data to determine this information.  We have also created an additional table (Table 2) to highlight this information.

 8. Conclusion: the content of the conclusion paragraph would fit more for an introduction rather than a wrap-up of the main findings. I suggest to significantly modify this section, in order to briefly mention the novel agents in clinical development and their selective advantages compared to our current antifungal armamentarium.

**Author response**:  We have modified the conclusion as suggested by the reviewer to summarize and highlight the advantages of the most promising investigational agents discussed in the manuscript.
